# A case–control study of selenium in nails and prostate cancer risk in British men

**DOI:** 10.1038/sj.bjc.6601701

**Published:** 2004-03-02

**Authors:** N E Allen, J S Morris, R A Ngwenyama, T J Key

**Affiliations:** 1 Cancer Research UK Epidemiology Unit, University of Oxford, Gibson Building, Radcliffe Infirmary, Oxford OX2 6HE, UK; 2 University of Missouri-Columbia, Research Reactor Center, Columbia, MO 65211, USA

**Keywords:** selenium, nails, prostate cancer, epidemiology

## Abstract

In view of the experimental evidence suggesting that the micronutrient selenium reduces prostate cancer risk, we investigated the association between the selenium level in fingernails, a measure of long-term selenium intake, and prostate cancer risk in a case–control study among 656 British men, conducted in 1989–1992. Nail clippings were taken at the time of recruitment and selenium concentration, measured using neutron activation techniques, was successfully assayed for 300 case–control pairs and varied six-fold among the controls (0.59 p.p.m.; interquartile range, 0.50–0.71 p.p.m.). Nail selenium concentration was not significantly associated with prostate cancer risk: men in the highest quartile of nail selenium had a slightly increased risk compared with men in the lowest quartile (OR 1.24, 95 CI, 0.73–2.10); for advanced prostate cancer, men in the highest quartile had a slightly reduced risk compared with men in the lowest quartile (OR 0.78, 95% CI, 0.27–2.25). These results suggest that selenium is not strongly associated with prostate cancer risk in British men.

Selenium is an essential trace element found largely in grains, fish and meat products and has been shown to have antioxidant and other potentially anticarcinogenic properties (Combs and Gray, 1998). Experimental evidence indicates that selenium supplementation can reduce the incidence of viral and chemically induced tumours in animals (Vernie, 1984), including prostate cancer cell lines (Menter *et al*, 2000).

The selenium content in foods varies widely between regions (Levander, 1987) because it is largely determined by the amount of selenium in the soil. Ecological data suggest that regional cancer mortality rates in the United States are inversely correlated with the selenium concentration in plants (Clark *et al*, 1991), and a randomised controlled trial among skin cancer patients found selenium supplementation of 200 *μ*g day^−1^ to be associated with a significant 63% reduction in prostate cancer incidence (Clark *et al*, 1996; Duffield-Lillico *et al*, 2002). However, little is known about the relationship between the selenium status and prostate cancer risk in the general population.

The aim of this study is to investigate the association between nail selenium concentration, a marker of long-term intake, and prostate cancer risk. A subsidiary aim is to examine whether factors that may influence selenium status, such as smoking (Hunter *et al*, 1990), vitamin E intake (Diplock, 1978) or the aggressiveness of disease at the time of recruitment (Yoshizawa *et al*, 1998; Goodman *et al*, 2001) modify the association between selenium concentration and prostate cancer risk.

## SUBJECTS AND METHODS

A population-based case–control study of the association between diet and prostate cancer was established in Britain, between 1989 and 1992, and included 328 men with incident prostate cancer and 328 population-based controls (Key *et al*, 1997). The study was restricted to white men under the age of 75 years who could speak English and who were well enough to complete a diet interview and give a reasonable history. Identification of cases was by searching appropriate medical records and cancer registries throughout Oxfordshire, West Berkshire and Leeds; the study was approved by the local ethics committees in these areas. The date of diagnosis was taken as the date on the histopathology report or the date of the first letter from the consultant giving a diagnosis of prostate cancer. Cases were classified as having advanced prostate cancer if there was radiological or histopathological evidence of local invasion and/or metastases to the bone or soft tissue. Controls were taken from the patient list of the general practitioner of each case and were matched on age (within 1 year) and region of residence. Men who had a previous diagnosis of prostate cancer or who had had a radical prostatectomy were not eligible as controls. One control was selected for each case, based either on the closest date of birth (for practices with computerised records) or on alphabetical order of surname after omitting the first 10 patients following the case (for practices without computerised records). Of the 425 eligible cases identified, 328 were interviewed (77.2%), 33 refused (7.8%), for 28 the general practitioner had advised against contact (6.6%), 34 had died before an interview could be obtained (8.0%) and two had emigrated outside the study area (0.5%). Of the first 328 controls selected, 267 agreed to be interviewed (81.4%), 42 refused (12.8%) and for 19 the consultant or general practitioner had advised against contact (5.8%). Informed written consent was obtained from each of the study subjects. All men were interviewed by one of three female interviewers, who also took fingernail clippings at the same time as completion of the interview. The same interviewer covered all the cases and controls in each of the three health districts. In total, 93% of cases were interviewed within 1 year of diagnosis (median interval of 4 months).

All participants completed a questionnaire that included details of age, anthropometric, smoking and other lifestyle factors as well as a detailed semiquantitative food-frequency questionnaire that assessed intake of 83 food items during the last 5 years. Study participants were invited to contribute fingernail clippings, of which 632 (96%) did so. Nail clippings were kept in an envelope marked with a unique study identification number and stored at room temperature until analysis. The selenium content was assayed using instrumental neutron activation analysis techniques at the University of Missouri-Columbia Research Reactor Center, Columbia, USA. Case and control specimens were analysed within the same batch in random order and laboratory personnel were blinded to the case–control status of each individual. Nail clippings were thoroughly washed with de-ionised water and freeze-dried. Specimens were irradiated with neutrons and transferred to a high-resolution gamma-ray spectrometer where the gamma-ray from the decay of ^77m^Se was quantified and selenium concentrations were determined by standard comparison. For 94 subjects, there was adequate sample to prepare and analyse at least two replicate subsamples to check sample homogeneity and analytical reproducibility. A mean and standard deviation was calculated for each replicate. The average relative standard deviation of the mean for these 94 replicate sets was 4.1%. A Standard Reference Material, supplied by the National Institutes of Standards and Technology (NIST SRM 1577 Bovine Liver) having a certified selenium concentration (1.1±0.1 p.p.m.) was analysed with each sample batch. In total, 24 aliquots of NIST SRM 1577 were analysed giving a relative standard deviation of the mean of 3.3%.

### Statistical analysis

Since the distributions of nail selenium values and dietary intakes were skewed toward the higher levels, these values were naturally logarithmically transformed to approximate a normal distribution. The means and their corresponding 95 percent confidence intervals (95% CI) are presented as back-transformed values. Comparisons of the geometric mean nail selenium concentration by sociodemographic and lifestyle variables were examined using analysis of variance. Comparison of the mean selenium concentration by case–control status was examined using the paired *t*-test and analysis of variance, where appropriate. Selenium values were categorised into fourths based on the distribution among the control subjects and the association between nutrient intake and selenium concentration was assessed using one-way analysis of variance. Relative risks were estimated as odds ratios (with their corresponding 95% CIs) using conditional logistic regression techniques and potential confounders were included as covariates in the models. Odds ratios for each quartile were calculated using the lowest fourth as the referent category. Where appropriate, a test for linear trend was performed to assess statistical significance across exposure categories by including the median value of each quartile among the controls as a continuous variable in the models. All *P*-values were based on two-tailed tests and a value of less than 0.05 was considered statistically significant. Interaction effects were examined between the selenium status and the aggressiveness of the disease, smoking and vitamin E intake. For the latter analysis, selenium concentration and vitamin E intake were categorised into two groups according to the median concentration among the controls. Interaction effects were assessed by the likelihood ratio test for the interaction term added to a model that also included the variables as separate factors. All analyses were conducted using Stata version 8.0 (Statacorp, 2003).

## RESULTS

Out of the 656 study participants, 24 subjects did not provide nail samples (17 cases; seven controls) and five subjects had selenium values that remained very high after repeated laboratory analysis (range 4.6–35.1 p.p.m.) and were thus excluded from subsequent statistical analysis (one case; four controls). In total, 300 case–control pairs were available for analysis.

The mean age at the time of recruitment was 68.6 for cases and 68.3 for controls and ranged from 44 to 77 years. Among the 300 controls, selenium values ranged six-fold from 0.354 to 2.102 p.p.m. with a median concentration of 0.593 p.p.m. and an interquartile range of 0.498–0.705 p.p.m. Table 1

**Table 1:** Geometric mean selenium concentration according to baseline characteristics among 300 controls

**Variable**	**Number**	**Mean Se concentration**	***P*-value**
*Age (years)*			
<60	17	0.690	
60–69	124	0.600	
70+	159	0.612	0.15
			
*Region*			
Oxfordshire	102	0.643	
West Berkshire	109	0.614	
Leeds	89	0.573	<0.0001
			
*BMI at age 25 years (kg m*^*−2*^*)*^a^			
<21.3	112	0.606	
21.3–23.3	91	0.625	
23.4+	96	0.601	0.63
			
*BMI at age 45 years (kg m*^*−2*^*)*^b^			
<23.0	110	0.604	
23.0–25.3	97	0.626	
25.4+	91	0.603	0.60
			
*Smoking*			
Never	74	0.643	
Past	175	0.615	
Current	51	0.556	0.01
			
*Cigarettes day*^*−1*^^c^			
<20	24	0.599	
20+	27	0.520	0.06
			
*Social class*			
I, II, III nonmanual	167	0.624	
III manual, IV, V	133	0.595	0.15

a One control had missing data.

b Two controls had missing data.

c Among current smokers only.

shows the geometric mean selenium concentration according to baseline characteristics among the 300 control subjects. The mean nail selenium concentration differed significantly by region, being highest in men recruited from Oxfordshire and lowest in men recruited from the Leeds region (test for heterogeneity; *P*<0.0001). However, adjustment for age group, smoking status and total energy intake reduced the differences in nail selenium concentration among the three regions (*P*=0.06; results not shown). Smoking was significantly associated with nail selenium concentration, with current smokers having, on average, a 14% lower selenium concentration than never smokers and a 10% lower concentration than past smokers (test for heterogeneity: *P*=0.014). Among current smokers, men who smoked 20 cigarettes or more per day had 13% lower levels than men who smoked less than 20 cigarettes per day, although this difference was not statistically significant (*P*=0.06). Age, body mass index (BMI; weight (kg)/height (m)^2^) at ages 25 and 45 years and social class were not associated with nail selenium concentration.

Table 2

**Table 2:** Association between baseline characteristics and prostate cancer risk

	**Cases**	**Controls**	**Odds ratio**
	**(*n*=300)**	**(*n*=300)**	**(95% CI)**
*Marital status*^a^			
Married	228	245	1.00
Unmarried	72	55	1.40 (0.95–2.09)
Test for heterogeneity			*P*=0.11
			
*Smoking status*			
Never	75	74	1.00
Past	175	175	0.99 (0.66–1.47)
Current	50	51	0.97 (0.59–1.59)
Test for heterogeneity			*P*=0.90
			
*Family history of prostate cancer*^b^			
No	285	293	1.00
Yes	15	7	2.14 (0.87–5.26)
Test for heterogeneity			*P*=0.10
			
*Social class*			
I,II,III nonmanual	139	167	1.00
III manual, IV, V	161	133	1.54 (1.09–2.18)
Test for heterogeneity			*P*=0.02
			
*BMI at age 25 years (kg m*^*−2*^*)*			
<21.3	94	112	1.00
21.3–23.3	113	91	1.45 (0.98–2.15)
23.4+	91	96	1.14 (0.77–1.69)
Test for linear trend			*P*=0.53
			
*BMI at age 45 years (kg m*^*−2*^*)*			
<23.0	91	110	1.00
23.0–25.3	97	97	1.19 (0.80–1.73)
25.4+	110	91	1.42 (0.97–2.08)
Test for linear trend			*P*=0.07

a Married refers to married or cohabitating men; unmarried refers to single, separated, divorced or widowed men.

b Reporting having had a father or brother with prostate cancer.

shows the association of baseline characteristics with cancer risk among 300 prostate cancer cases and their controls. Social class was significantly associated with prostate cancer risk; men in the lower social class group had a 54% increased risk of developing prostate cancer compared with men in a higher social class group (OR 1.54; 95% CI, 1.09–2.18). The proportion of men with a family history of prostate cancer was higher among cases than controls, although the association was not statistically significant (OR 2.14; 95% CI, 0.87–5.26). Marital status, smoking and BMI at age 25 or 45 years were not associated with risk. These associations are similar to those previously reported in the full case–control study of 656 subjects (Key *et al*, 1997).

There was no significant difference in the mean selenium concentration between cases and controls; the geometric mean was 0.622 p.p.m. in cases and 0.611 p.p.m. in controls (*P*=0.454, Table 3

**Table 3:** Geometric mean nail selenium concentrations in cases and controls

	**Cases**		**Controls**		
	**Number**	**Mean**	**Number**	**Mean**	***P*-value**
*Total cancer*	300	0.622	300	0.611	0.45
Localised	211	0.631	211	0.607	0.17
Advanced	89	0.602	89	0.621	0.47

); adjustment for social class made no appreciable difference to the means (results not shown). There was also no significant difference between the geometric mean selenium concentration in men with localised or advanced cancer and that of their controls (Table 3), or between men with localised and advanced disease (*P*=0.215).

Details of risk by quartile of nail selenium concentration are shown in Table 4

**Table 4:** Association of nail selenium concentration with prostate cancer risk^a^

**Quartile of nail selenium**	**Quartile median (p.p.m.)**	**Total prostate cancer (*n* =300 cases)**		**Localised cancer (*n*=211 cases)**		**Advanced cancer (*n*=89 cases)**	
<0.497	0.456	62	1.00	39	1.00	23	1.00
0.497–0.592	0.547	84	1.39 (0.87–2.23)	61	1.54 (0.85–2.77)	23	0.99 (0.43–2.26)
0.593–0.704	0.635	80	1.25 (0.78–2.02)	57	1.59 (0.90–2.82)	23	0.65 (0.26–1.56)
0.705+	0.837	74	1.24 (0.73–2.10)	54	1.45 (0.78–2.70)	20	0.78 (0.27–2.25)
Test for linear trend			*P*=0.581		*P*=0.313		*P*=0.480

a All results are matched for age and region and adjusted for social class.

: overall, no association was found, either before or after adjustment for social class. Men in the highest quartile had a nonsignificant increased risk compared with men in the lowest quartile (OR 1.24; 95% CI, 0.73–2.10). Additional adjustment for smoking made no difference to the association between selenium concentration and risk and the association was similar when current smokers were excluded from the analysis (results not shown). The test for multiplicative interaction between selenium concentration (in quartiles) and smoking status was not statistically significant after including these terms together with the interaction term into the model (*P*=0.169). Additional adjustment for food items and nutrients found to be associated with prostate cancer risk in our study of a previous analysis of this study population, including garlic, beans, peas and vitamin B_6_ intake (Key *et al*, 1997) made no appreciable difference to the risk estimates and were not included in the final model.

Selenium concentration was not associated with localised or advanced disease (Table 4). However, men in the highest quartile of selenium nail concentration had a non-significant 22% reduced risk of developing advanced prostate cancer compared with men in the lowest selenium quartile (OR=0.78; 95% CI, 0.27–2.25).

There was no evidence that vitamin E intake was independently associated with risk, either before or after adjustment for social class (OR for vitamin E intake of 12 or more mg day^−1^ *vs* less than 12 mg day^−1^=0.85; 95% CI, 0.62–1.17), and there was no evidence of interaction with selenium concentration on risk (*P*=0.729).

## DISCUSSION

The findings from this study suggest that selenium concentration, as measured in fingernail clippings, is not strongly associated with prostate cancer risk. Although previous studies have assessed the selenium content in toe nails, which is 9–15% lower than that of fingernails (Baskett *et al*, 1995), selenium concentrations in these British men are still somewhat lower than those reported in North American populations (Yoshizawa *et al*, 1998; Ghadirian *et al*, 2000; Helzlsouer *et al*, 2000) and are comparable with other European populations (Kardinaal *et al*, 1997; van den Brandt *et al*, 2003). It is well established that the selenium content of European soils is low and that dietary intake has been gradually declining due to a reduction in imported selenium-rich wheat from the US and Canada, together with a general decline in cereal consumption (Rayman, 1997). Indeed, it is estimated that selenium intake in the UK has declined by about a third from an average of 60 *μ*g day^−1^ in the late 1970s to 30–40 *μ*g day^−1^ in the late 1990s, which is approximately half the reference daily intake of 75 *μ*g day^−1^ for adult men (Rayman, 1997). Although dietary selenium intake was not estimated in this study population because of the wide variation in soil selenium content and the lack of information about supplemental selenium use, the low nail selenium concentrations confirm that dietary intake in British men is indeed lower than that of typical North American populations.

Our finding that current smokers have lower selenium levels than never-smokers is consistent with previous studies (Hunter *et al*, 1990; Swanson *et al*, 1990; van den Brandt *et al*, 1993). However, smoking was not associated with risk in this study population and additional adjustment for smoking made no appreciable difference to the association between selenium concentration and cancer risk.

The hypothesis that selenium and vitamin E intake may interact to influence prostate cancer risk has been largely derived from animal studies (Diplock, 1978) and from a randomised controlled trial that found *α*-tocopherol to be associated with a reduced risk of prostate cancer (Heinonen *et al*, 1998). Indeed, a trial specifically designed to test the joint efficacy of selenium and vitamin E for the prevention of prostate cancer is underway (Klein *et al*, 2003). Evidence from observational studies has, however, been inconsistent; although one study found that risk was lowest among men with both a high selenium and *δ*-tocopherol level (Helzlsouer *et al*, 2000), the present study and others have found no interaction of vitamin E intake and selenium levels with risk (Hartman *et al*, 1998; Yoshizawa *et al*, 1998; Vogt *et al*, 2003).

The main strengths of this study are the large number of cases and controls, the ability to categorise cancers into localised and advanced disease, and detailed information on potential confounding factors. Further, selenium measures based on nail samples have been shown to reflect selenium intake integrated over the previous 6–12 months (Morris *et al*, 1983) and can be used to rank subjects according to long-term dietary selenium intake (Longnecker *et al*, 1996). Moreover, a single measure has shown moderate reliability over a 6-year period, with a correlation coefficient of 0.48 (Garland *et al*, 1993).

The main limitation of this study is the possibility that the cancer or its treatment may affect nutritional status and thereby selenium levels. However, as nails reflect nutritional status for up to a year prior to clipping, any recent changes in nutrient intake or physiology are unlikely to influence nail concentration over the short-term. The observation that the mean selenium concentration was similar between men with localised and advanced disease also suggests that disease status is unlikely to influence the association between selenium concentration and prostate cancer risk in this study population.

In conclusion, the findings from this study suggest that selenium concentration, as measured in nail clippings, is not strongly associated with prostate cancer risk in British men. This is in contrast to the results of the Nutritional Prevention of Cancer trial that found selenium supplementation to be associated with a significant reduction in prostate cancer risk (Clark *et al*, 1996; Duffield-Lillico *et al*, 2002). Several prospective studies have also found significant protective associations for serum or nail selenium levels in relation to prostate cancer (Yoshizawa *et al*, 1998; Helzlsouer *et al*, 2000; Nomura *et al*, 2000; Brooks *et al*, 2001; van den Brandt *et al*, 2003). However, consistent with the findings from the present study, some other observational studies have failed to find such an association (Willett *et al*, 1983; Coates *et al*, 1988; Knekt *et al*, 1990; Hardell *et al*, 1995; Ghadirian *et al*, 2000; Goodman *et al*, 2001; Vogt *et al*, 2003). It is unclear whether these epidemiological data overall are or are not supportive of a reduction in prostate cancer risk with high selenium intake, and a pooled analysis might be informative.
